# 2-[(*E*)-Phen­yl(2-phenyl­hydrazin-1-yl­idene)meth­yl]phenol

**DOI:** 10.1107/S1600536812005387

**Published:** 2012-02-24

**Authors:** R. Alan Howie, James L. Wardell, Solange M. S. V. Wardell, Edward R. T. Tiekink

**Affiliations:** a Department of Chemistry, University of Aberdeen, Meston Walk, Old Aberdeen AB24 3UE, Scotland; b Centro de Desenvolvimento Tecnológico em Saúde (CDTS), Fundação Oswaldo Cruz (FIOCRUZ), Casa Amarela, Campus de Manguinhos, Avenida Brasil 4365, 21040-900, Rio de Janeiro, RJ, Brazil; c CHEMSOL, 1 Harcourt Road, Aberdeen AB15 5NY, Scotland; d Department of Chemistry, University of Malaya, 50603 Kuala Lumpur, Malaysia

## Abstract

In the title hydrazone derivative, C_19_H_16_N_2_O, a twist is found between the hy­droxy­phenyl and *N*-bound phenyl rings [dihedral angle = 24.37 (7)°]. The C-bound phenyl ring is almost perpendicular to each of these planes [dihedral angles = 75.30 (7) and 86.00 (7)°, respectively]. The conformation about the imine bond [1.2935 (17) Å] is *E*. The hy­droxy group forms an intra­molecular hydrogen bond with the imine N atom. Zigzag chains along [001] mediated by N—H⋯O hydrogen bonds feature in the crystal packing.

## Related literature

For background on the influence of substituents upon the supra­molecular structures of hydrazones, see: Glidewell *et al.* (2004 ▶); Ferguson *et al.* (2005 ▶); Wardell *et al.* (2007 ▶); Baddeley, de Souza França *et al.* (2009 ▶); Baddeley, Howie *et al.* (2009 ▶); de Souza *et al.* (2010 ▶); Howie, da Silva Lima *et al.* (2010 ▶); Howie, de Souza *et al.* (2010 ▶); Nogueira *et al.* (2011 ▶); Howie *et al.* (2011 ▶).
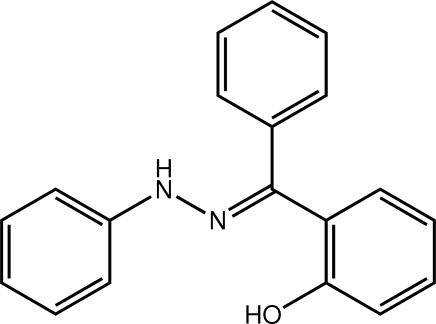


## Experimental

### 

#### Crystal data

C_19_H_16_N_2_O
*M* *_r_* = 288.34
Monoclinic, 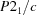

*a* = 9.6796 (3) Å
*b* = 15.3312 (5) Å
*c* = 10.3593 (2) Å
β = 108.149 (2)°
*V* = 1460.84 (7) Å^3^
*Z* = 4
Mo *K*α radiation
μ = 0.08 mm^−1^
*T* = 120 K
0.49 × 0.38 × 0.18 mm

#### Data collection

Bruker–Nonius Roper CCD camera on κ-goniostat diffractometer
Absorption correction: multi-scan (*SADABS*; Sheldrick, 2007 ▶) *T*_min_ = 0.857, *T*_max_ = 0.985
16532 measured reflections
3337 independent reflections
2624 reflections with *I* > 2σ(*I*)
*R*_int_ = 0.044

#### Refinement

*R*[*F* ^2^ > 2σ(*F* ^2^)] = 0.051
*wR*(*F* ^2^) = 0.141
*S* = 1.06
3337 reflections
205 parameters
2 restraints
H atoms treated by a mixture of independent and constrained refinement
Δρ_max_ = 0.31 e Å^−3^
Δρ_min_ = −0.40 e Å^−3^

### 

Data collection: *COLLECT* (Hooft, 1998 ▶); cell refinement: *DENZO* (Otwinowski & Minor, 1997 ▶) and *COLLECT*; data reduction: *DENZO* and *COLLECT*; program(s) used to solve structure: *SHELXS97* (Sheldrick, 2008 ▶); program(s) used to refine structure: *SHELXL97* (Sheldrick, 2008 ▶); molecular graphics: *ORTEP-3* (Farrugia, 1997 ▶) and *DIAMOND* (Brandenburg, 2006 ▶); software used to prepare material for publication: *publCIF* (Westrip, 2010 ▶).

## Supplementary Material

Crystal structure: contains datablock(s) global, I. DOI: 10.1107/S1600536812005387/bt5814sup1.cif

Structure factors: contains datablock(s) I. DOI: 10.1107/S1600536812005387/bt5814Isup2.hkl

Supplementary material file. DOI: 10.1107/S1600536812005387/bt5814Isup3.cml

Additional supplementary materials:  crystallographic information; 3D view; checkCIF report

## Figures and Tables

**Table 1:** Hydrogen-bond geometry (Å, °)

*D*—H⋯*A*	*D*—H	H⋯*A*	*D*⋯*A*	*D*—H⋯*A*
O1—H1O⋯N1	0.85 (1)	1.76 (1)	2.5678 (14)	157 (2)
N2—H2N⋯O1^i^	0.89 (2)	2.43 (2)	3.2517 (16)	155 (1)

Symmetry code: (i) 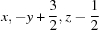.

**Fig. 1.**
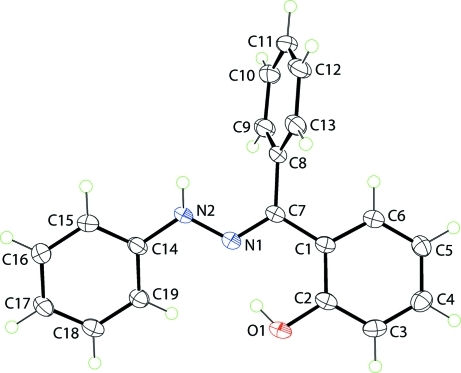
The molecular structure of (I) showing the atom-labelling scheme and displacement ellipsoids at the 50% probability level.

**Fig. 2.**
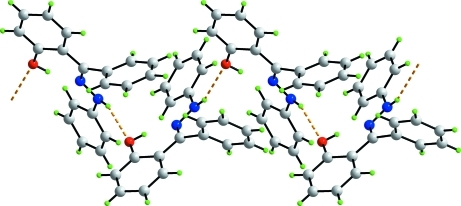
A view of the supramolecular zigzag chain in (I) sustained by N—H···O (orange dashed lines) hydrogen bonds.

**Fig. 3.**
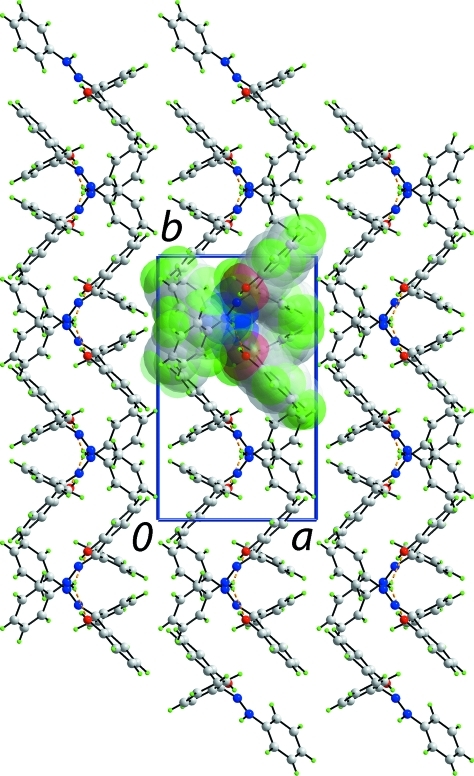
A view in projection down the c axis of the packing of supramolecular chains in (I). The N—H···O hydrogen bonds are shown as orange dashed lines. One chain is highlighted in space-filling mode.

## supplementary crystallographic information

### Comment

For some time, we have been interested in the influence of substituents upon the supramolecular structures of hydrazones, especially of those having potential biological activities. These include substituted phenylhydrazines with substituted benzaldehydes (Glidewell *et al.*, 2004; Ferguson *et al.*, 2005) and 2-hydroxyacetophenone (Baddeley, de Souza França *et al.*, 2009). Hydrazones derived from substituted benzaldehydes and (pyrazinecarbonyl)hydrazine (Baddeley, Howie *et al.*, 2009; Howie, da Silva Lima *et al.*, 2010), 2-hydrazinyl-benzothiazole (Nogueira *et al.*, 2011), 7-chloroquinoline-4-hydrazide (Howie, de Souza *et al.*, 2010; de Souza *et al.*, 2010) and 2-hydrazinylacyl-*N*-isonicotine (Wardell *et al.*, 2007) have also been investigated along with *L*-serinyl derivatives, (*S*)-2-hydroxy-1-[*N*-(benzylidene)-hydrazinylcarbonyl]ethylcarbamate esters (Howie *et al.*, 2011). In continuation of these studies, herein the crystal and molecular structure of (*E*)-2-hydroxybenzophenone phenylhydrazone (I) is described.

In (I), Fig. 1, the hydroxy-benzene and *N*-bound phenyl rings are twisted, forming a dihedral angle of 24.37 (7)°. These planes form dihedral angles of 75.30 (7) and 86.00 (7)°, respectively, with the *C*-bound phenyl ring indicating an almost perpendicular relationship. The hydroxy group forms an intramolecular hydrogen bond with the imine-N1 atom, Table 1. The configuration about the imine bond N1═C7 [1.2935 (17) Å] is *E*.

The most prominent feature of the crystal packing is the formation of zigzag chains along [001] generated by glide symmetry and mediated by N—H···O hydrogen bonds, Fig. 2 and Table 1. Chains pack in the crystal structure with no specific intermolecular interactions between them, Fig. 3.

### Experimental

A solution of phenylhydrazine and 2-hydroxybenzophenone (1 mmol each) in ethanol (20 ml) was refluxed for 1 h, rotary evaporated and the residue recrystallized from ethanol. IR (KBr, cm^-1^): ν 3302, 1600, 1557. Analysis found: C 78.78, H 5.81, N 9.47%; calculated for C_19_H_16_N_2_O: C 79.14, H 5.59, N 9.71%.

### Refinement

The C-bound H atoms were geometrically placed (C—H = 0.95 Å) and refined as riding with *U*_iso_(H) = 1.2*U*_eq_(C). The O- and N-bound H atoms were located from a difference map and refined with the distance restraints O—H = 0.84±0.01 and N—H = 0.88±0.01 Å, and with *U*_iso_(H) = *zU*_eq_(carrier atom); *z* = 1.5 for O and *z* = 1.2 for N.

### Crystal data

**Table:**

C_19_H_16_N_2_O	*F*(000) = 608
*M**_r_* = 288.34	*D*_x_ = 1.311 Mg m^−^^3^
Monoclinic, *P*2_1_/*c*	Mo *K*α radiation, λ = 0.71073 Å
Hall symbol: -P 2ybc	Cell parameters from 9588 reflections
*a* = 9.6796 (3) Å	θ = 2.9–27.5°
*b* = 15.3312 (5) Å	µ = 0.08 mm^−^^1^
*c* = 10.3593 (2) Å	*T* = 120 K
β = 108.149 (2)°	Slab, yellow
*V* = 1460.84 (7) Å^3^	0.49 × 0.38 × 0.18 mm
*Z* = 4	

### Data collection

**Table:**

Bruker–Nonius Roper CCD camera on κ-goniostat diffractometer	3337 independent reflections
Radiation source: Bruker–Nonius FR591 rotating anode	2624 reflections with *I* > 2σ(*I*)
Graphite monochromator	*R*_int_ = 0.044
Detector resolution: 9.091 pixels mm^-1^	θ_max_ = 27.5°, θ_min_ = 3.4°
φ and ω scans	*h* = −12→12
Absorption correction: multi-scan (*SADABS*; Sheldrick, 2007)	*k* = −19→19
*T*_min_ = 0.857, *T*_max_ = 0.985	*l* = −13→13
16532 measured reflections	

### Refinement

**Table:**

Refinement on *F*^2^	Primary atom site location: structure-invariant direct methods
Least-squares matrix: full	Secondary atom site location: difference Fourier map
*R*[*F*^2^ > 2σ(*F*^2^)] = 0.051	Hydrogen site location: inferred from neighbouring sites
*wR*(*F*^2^) = 0.141	H atoms treated by a mixture of independent and constrained refinement
*S* = 1.06	*w* = 1/[σ^2^(*F*_o_^2^) + (0.0846*P*)^2^ + 0.1552*P*] where *P* = (*F*_o_^2^ + 2*F*_c_^2^)/3
3337 reflections	(Δ/σ)_max_ < 0.001
205 parameters	Δρ_max_ = 0.31 e Å^−^^3^
2 restraints	Δρ_min_ = −0.40 e Å^−^^3^

### Special details

**Table:**

Geometry. All s.u.'s (except the s.u. in the dihedral angle between two l.s. planes) are estimated using the full covariance matrix. The cell s.u.'s are taken into account individually in the estimation of s.u.'s in distances, angles and torsion angles; correlations between s.u.'s in cell parameters are only used when they are defined by crystal symmetry. An approximate (isotropic) treatment of cell s.u.'s is used for estimating s.u.'s involving l.s. planes.
Refinement. Refinement of *F*^2^ against ALL reflections. The weighted *R*-factor *wR* and goodness of fit *S* are based on *F*^2^, conventional *R*-factors *R* are based on *F*, with *F* set to zero for negative *F*^2^. The threshold expression of *F*^2^ > 2σ(*F*^2^) is used only for calculating *R*-factors(gt) *etc*. and is not relevant to the choice of reflections for refinement. *R*-factors based on *F*^2^ are statistically about twice as large as those based on *F*, and *R*- factors based on ALL data will be even larger.

### Fractional atomic coordinates and isotropic or equivalent isotropic displacement parameters (Å^2^)

**Table:**

	*x*	*y*	*z*	*U*_iso_*/*U*_eq_	
O1	0.55543 (11)	0.62061 (7)	0.67432 (9)	0.0305 (3)	
H1O	0.5167 (19)	0.6457 (11)	0.5981 (12)	0.046*	
N1	0.49781 (12)	0.67646 (7)	0.42953 (11)	0.0245 (3)	
N2	0.42408 (13)	0.73657 (8)	0.33672 (11)	0.0279 (3)	
H2N	0.4722 (16)	0.7602 (10)	0.2854 (14)	0.033*	
C1	0.68370 (14)	0.57283 (9)	0.51935 (12)	0.0219 (3)	
C2	0.65661 (15)	0.56890 (9)	0.64615 (13)	0.0245 (3)	
C3	0.73482 (15)	0.51112 (9)	0.74555 (13)	0.0275 (3)	
H3	0.7182	0.5098	0.8312	0.033*	
C4	0.83577 (16)	0.45602 (9)	0.72145 (14)	0.0294 (3)	
H4	0.8872	0.4163	0.7900	0.035*	
C5	0.86344 (16)	0.45784 (9)	0.59745 (14)	0.0268 (3)	
H5	0.9333	0.4196	0.5808	0.032*	
C6	0.78782 (15)	0.51608 (9)	0.49900 (13)	0.0245 (3)	
H6	0.8073	0.5176	0.4146	0.029*	
C7	0.60591 (14)	0.63406 (8)	0.41159 (12)	0.0225 (3)	
C8	0.65557 (15)	0.64475 (9)	0.28938 (13)	0.0233 (3)	
C9	0.57166 (16)	0.61134 (10)	0.16460 (13)	0.0295 (3)	
H9	0.4805	0.5848	0.1559	0.035*	
C10	0.62186 (17)	0.61703 (10)	0.05272 (14)	0.0332 (4)	
H10	0.5659	0.5929	−0.0319	0.040*	
C11	0.75214 (18)	0.65735 (10)	0.06393 (14)	0.0342 (4)	
H11	0.7858	0.6608	−0.0128	0.041*	
C12	0.83445 (17)	0.69288 (10)	0.18688 (15)	0.0331 (4)	
H12	0.9230	0.7221	0.1939	0.040*	
C13	0.78680 (16)	0.68548 (9)	0.29963 (14)	0.0286 (3)	
H13	0.8444	0.7084	0.3845	0.034*	
C14	0.31426 (15)	0.78382 (9)	0.36540 (12)	0.0246 (3)	
C15	0.26578 (17)	0.86137 (10)	0.29652 (14)	0.0335 (4)	
H15	0.3084	0.8820	0.2312	0.040*	
C16	0.15586 (17)	0.90856 (11)	0.32279 (15)	0.0362 (4)	
H16	0.1232	0.9613	0.2749	0.043*	
C17	0.09268 (16)	0.87973 (10)	0.41823 (14)	0.0319 (4)	
H17	0.0173	0.9124	0.4362	0.038*	
C18	0.14125 (15)	0.80271 (10)	0.48685 (14)	0.0282 (3)	
H18	0.0990	0.7827	0.5528	0.034*	
C19	0.25048 (15)	0.75439 (9)	0.46098 (13)	0.0253 (3)	
H19	0.2820	0.7013	0.5082	0.030*	

### Atomic displacement parameters (Å^2^)

**Table:**

	*U*^11^	*U*^22^	*U*^33^	*U*^12^	*U*^13^	*U*^23^
O1	0.0325 (6)	0.0394 (6)	0.0258 (5)	0.0046 (4)	0.0178 (4)	−0.0006 (4)
N1	0.0247 (6)	0.0277 (6)	0.0239 (5)	0.0032 (5)	0.0118 (5)	0.0002 (4)
N2	0.0281 (7)	0.0352 (7)	0.0262 (6)	0.0094 (5)	0.0171 (5)	0.0058 (5)
C1	0.0214 (7)	0.0247 (7)	0.0218 (6)	−0.0030 (5)	0.0098 (5)	−0.0021 (5)
C2	0.0230 (7)	0.0287 (7)	0.0250 (6)	−0.0044 (5)	0.0122 (5)	−0.0049 (5)
C3	0.0298 (8)	0.0326 (8)	0.0222 (6)	−0.0068 (6)	0.0112 (5)	−0.0002 (6)
C4	0.0298 (8)	0.0279 (7)	0.0299 (7)	−0.0031 (6)	0.0086 (6)	0.0047 (6)
C5	0.0258 (7)	0.0236 (7)	0.0329 (7)	0.0014 (5)	0.0117 (6)	−0.0011 (6)
C6	0.0265 (7)	0.0251 (7)	0.0246 (6)	−0.0029 (5)	0.0120 (5)	−0.0025 (5)
C7	0.0225 (7)	0.0250 (7)	0.0229 (6)	−0.0002 (5)	0.0115 (5)	−0.0020 (5)
C8	0.0250 (7)	0.0248 (7)	0.0238 (6)	0.0054 (5)	0.0130 (5)	0.0016 (5)
C9	0.0269 (8)	0.0367 (8)	0.0272 (7)	0.0016 (6)	0.0120 (6)	−0.0014 (6)
C10	0.0355 (9)	0.0428 (9)	0.0235 (7)	0.0085 (7)	0.0124 (6)	0.0001 (6)
C11	0.0452 (9)	0.0344 (8)	0.0323 (7)	0.0119 (7)	0.0256 (7)	0.0080 (6)
C12	0.0353 (9)	0.0301 (8)	0.0442 (8)	0.0019 (6)	0.0274 (7)	0.0032 (6)
C13	0.0286 (8)	0.0283 (7)	0.0330 (7)	0.0018 (6)	0.0156 (6)	−0.0020 (6)
C14	0.0225 (7)	0.0314 (7)	0.0218 (6)	0.0029 (6)	0.0094 (5)	−0.0035 (5)
C15	0.0356 (9)	0.0429 (9)	0.0278 (7)	0.0102 (7)	0.0181 (6)	0.0083 (6)
C16	0.0359 (9)	0.0428 (9)	0.0347 (8)	0.0160 (7)	0.0180 (7)	0.0112 (7)
C17	0.0266 (8)	0.0409 (9)	0.0320 (7)	0.0093 (6)	0.0147 (6)	0.0006 (6)
C18	0.0254 (8)	0.0346 (8)	0.0291 (7)	−0.0012 (6)	0.0151 (6)	−0.0023 (6)
C19	0.0248 (7)	0.0263 (7)	0.0271 (7)	−0.0004 (5)	0.0114 (6)	−0.0007 (5)

### Geometric parameters (Å, º)

**Table:**

O1—C2	1.3605 (17)	C9—C10	1.3920 (19)
O1—H1O	0.853 (9)	C9—H9	0.9500
N1—C7	1.2935 (17)	C10—C11	1.376 (2)
N1—N2	1.3621 (16)	C10—H10	0.9500
N2—C14	1.3928 (17)	C11—C12	1.386 (2)
N2—H2N	0.886 (9)	C11—H11	0.9500
C1—C6	1.3963 (19)	C12—C13	1.3875 (19)
C1—C2	1.4186 (17)	C12—H12	0.9500
C1—C7	1.4734 (18)	C13—H13	0.9500
C2—C3	1.390 (2)	C14—C15	1.391 (2)
C3—C4	1.372 (2)	C14—C19	1.3950 (19)
C3—H3	0.9500	C15—C16	1.382 (2)
C4—C5	1.3917 (19)	C15—H15	0.9500
C4—H4	0.9500	C16—C17	1.387 (2)
C5—C6	1.3812 (19)	C16—H16	0.9500
C5—H5	0.9500	C17—C18	1.383 (2)
C6—H6	0.9500	C17—H17	0.9500
C7—C8	1.4966 (17)	C18—C19	1.3838 (19)
C8—C13	1.390 (2)	C18—H18	0.9500
C8—C9	1.3924 (19)	C19—H19	0.9500
			
C2—O1—H1O	101.7 (13)	C8—C9—H9	120.1
C7—N1—N2	120.53 (11)	C11—C10—C9	120.41 (13)
N1—N2—C14	117.92 (10)	C11—C10—H10	119.8
N1—N2—H2N	116.2 (11)	C9—C10—H10	119.8
C14—N2—H2N	119.7 (11)	C10—C11—C12	120.21 (13)
C6—C1—C2	117.62 (12)	C10—C11—H11	119.9
C6—C1—C7	120.35 (11)	C12—C11—H11	119.9
C2—C1—C7	122.03 (12)	C13—C12—C11	119.65 (14)
O1—C2—C3	118.34 (12)	C13—C12—H12	120.2
O1—C2—C1	121.80 (12)	C11—C12—H12	120.2
C3—C2—C1	119.86 (13)	C12—C13—C8	120.53 (13)
C4—C3—C2	120.79 (12)	C12—C13—H13	119.7
C4—C3—H3	119.6	C8—C13—H13	119.7
C2—C3—H3	119.6	C15—C14—N2	119.55 (12)
C3—C4—C5	120.54 (13)	C15—C14—C19	119.18 (12)
C3—C4—H4	119.7	N2—C14—C19	121.27 (12)
C5—C4—H4	119.7	C16—C15—C14	120.26 (13)
C6—C5—C4	118.98 (13)	C16—C15—H15	119.9
C6—C5—H5	120.5	C14—C15—H15	119.9
C4—C5—H5	120.5	C15—C16—C17	120.74 (14)
C5—C6—C1	122.19 (12)	C15—C16—H16	119.6
C5—C6—H6	118.9	C17—C16—H16	119.6
C1—C6—H6	118.9	C18—C17—C16	118.93 (13)
N1—C7—C1	117.18 (11)	C18—C17—H17	120.5
N1—C7—C8	123.63 (12)	C16—C17—H17	120.5
C1—C7—C8	119.19 (11)	C19—C18—C17	121.03 (13)
C13—C8—C9	119.42 (12)	C19—C18—H18	119.5
C13—C8—C7	120.76 (12)	C17—C18—H18	119.5
C9—C8—C7	119.79 (12)	C18—C19—C14	119.87 (13)
C10—C9—C8	119.73 (14)	C18—C19—H19	120.1
C10—C9—H9	120.1	C14—C19—H19	120.1
			
C7—N1—N2—C14	−176.29 (12)	N1—C7—C8—C9	−72.19 (18)
C6—C1—C2—O1	−178.88 (12)	C1—C7—C8—C9	108.42 (15)
C7—C1—C2—O1	1.1 (2)	C13—C8—C9—C10	1.7 (2)
C6—C1—C2—C3	1.25 (19)	C7—C8—C9—C10	−176.44 (13)
C7—C1—C2—C3	−178.79 (12)	C8—C9—C10—C11	−1.6 (2)
O1—C2—C3—C4	178.47 (12)	C9—C10—C11—C12	−0.2 (2)
C1—C2—C3—C4	−1.7 (2)	C10—C11—C12—C13	1.8 (2)
C2—C3—C4—C5	1.0 (2)	C11—C12—C13—C8	−1.7 (2)
C3—C4—C5—C6	0.1 (2)	C9—C8—C13—C12	−0.1 (2)
C4—C5—C6—C1	−0.5 (2)	C7—C8—C13—C12	178.04 (12)
C2—C1—C6—C5	−0.2 (2)	N1—N2—C14—C15	161.37 (13)
C7—C1—C6—C5	179.83 (12)	N1—N2—C14—C19	−19.38 (19)
N2—N1—C7—C1	177.56 (11)	N2—C14—C15—C16	179.33 (14)
N2—N1—C7—C8	−1.8 (2)	C19—C14—C15—C16	0.1 (2)
C6—C1—C7—N1	171.04 (12)	C14—C15—C16—C17	0.3 (2)
C2—C1—C7—N1	−8.92 (19)	C15—C16—C17—C18	−0.1 (2)
C6—C1—C7—C8	−9.52 (19)	C16—C17—C18—C19	−0.4 (2)
C2—C1—C7—C8	170.52 (12)	C17—C18—C19—C14	0.7 (2)
N1—C7—C8—C13	109.69 (16)	C15—C14—C19—C18	−0.5 (2)
C1—C7—C8—C13	−69.71 (17)	N2—C14—C19—C18	−179.78 (12)

### Hydrogen-bond geometry (Å, º)

**Table:**

*D*—H···*A*	*D*—H	H···*A*	*D*···*A*	*D*—H···*A*
O1—H1*O*···N1	0.85 (1)	1.76 (1)	2.5678 (14)	157 (2)
N2—H2*N*···O1^i^	0.89 (2)	2.43 (2)	3.2517 (16)	155 (1)

Symmetry code: (i) *x*, −*y*+3/2, *z*−1/2.
